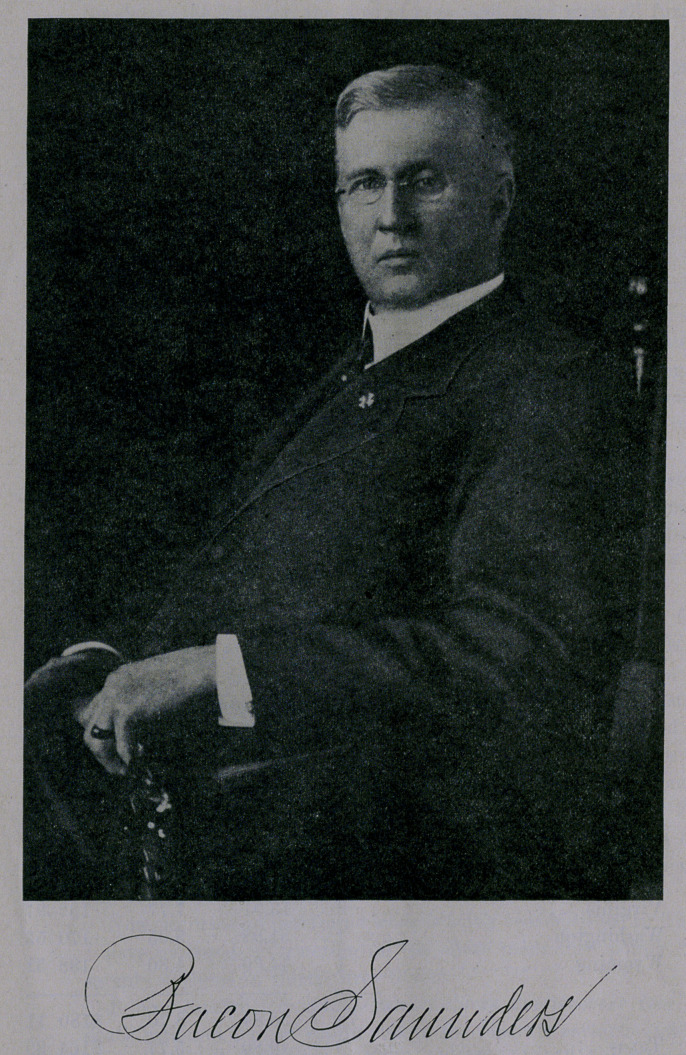# Dr. Bacon Saunders Honored

**Published:** 1915-01

**Authors:** 


Bacon Saunders

### Dr. Bacon Saunders Honored.

At the recent meeting held in Asheville, N. C., Dr. Bacon Saunders, of Fort Worth, was elected President of the Southern Surgical and Gynecological Association for the coming year. This is the most exclusive association in the United States, and Dr. Saunders has done his city, State and profession an honor in having merited this mark of distinction. The doctor is a native of Bowling Green, Ky., and was educated at Carleton College, Bonham, Texas. He graduated with the highest honors of his class from the Medical Department of the University of Louisville, afterwards taking post-graduate courses in New York, Philadelphia, Baltimore and other large clinical centers. He is Professor of Surgery and Clinical Surgery of the Fort Worth School of Medicine, Medical Department of Texas Christian University, and president of the faculty. He is also a member of the Board of Trustees of Texas Christian University. He is Chief Surgeon of the Fort Worth & Denver, the Wichita Valley and the Trinity & Brazos Valley Railways, and division surgeon for many of the railroads centering there. He is ex-President of the Texas State Medical Association, and ex-Vice-President of the American Association of Railway Surgeons. He is a fellow and one of the founders of the American College of Surgeons.

The Tarrant County Medical Society gave a banquet in compliment to Dr. Saunders, and over two hundred doctors from various parts of the State gathered to do honor to this great man.

A volume could be written on the life and achievements of this brave son of Texas, but his greatest claim to distinction is that, though he is at the head of his profession and always busy, he takes the time and has the courage and the inclination to help a professional brother who is less favored than he. He stands for the best and the highest in surgery and medicine, and it is eminently fitting that the choicest and highest honors should come to him.

Three cheers for the President of the Southern Surgical and Gynecological Association.

---

By a most unpardonable oversight, we failed to acknowledge our indebtedness to Wm. Wood & Co. for the loan of the illustrations that appeared in connection with Dr. Browning's article in the December number of the "Red Back." We are grateful for this courtesy, and regret that we failed to acknowledge it last month when the cuts appeared.

## Figures and Tables

**Figure f1:**